# Evolution of Taxis Responses in Virtual Bacteria: Non-Adaptive Dynamics

**DOI:** 10.1371/journal.pcbi.1000084

**Published:** 2008-05-23

**Authors:** Richard A. Goldstein, Orkun S. Soyer

**Affiliations:** 1 Mathematical Biology, National Institute for Medical Research, London, United Kingdom; 2 The Microsoft Research–University of Trento Centre for Computational and Systems Biology (CoSBi), Trento, Italy; University of Tokyo, Japan

## Abstract

Bacteria are able to sense and respond to a variety of external stimuli, with responses that vary from stimuli to stimuli and from species to species. The best-understood is chemotaxis in the model organism *Escherichia coli*, where the dynamics and the structure of the underlying pathway are well characterised. It is not clear, however, how well this detailed knowledge applies to mechanisms mediating responses to other stimuli or to pathways in other species. Furthermore, there is increasing experimental evidence that bacteria integrate responses from different stimuli to generate a coherent taxis response. We currently lack a full understanding of the different pathway structures and dynamics and how this integration is achieved. In order to explore different pathway structures and dynamics that can underlie taxis responses in bacteria, we perform a computational simulation of the evolution of taxis. This approach starts with a population of virtual bacteria that move in a virtual environment based on the dynamics of the simple biochemical pathways they harbour. As mutations lead to changes in pathway structure and dynamics, bacteria better able to localise with favourable conditions gain a selective advantage. We find that a certain dynamics evolves consistently under different model assumptions and environments. These dynamics, which we call non-adaptive dynamics, directly couple tumbling probability of the cell to increasing stimuli. Dynamics that are adaptive under a wide range of conditions, as seen in the chemotaxis pathway of *E. coli*, do not evolve in these evolutionary simulations. However, we find that stimulus scarcity and fluctuations during evolution results in complex pathway dynamics that result both in adaptive and non-adaptive dynamics depending on basal stimuli levels. Further analyses of evolved pathway structures show that effective taxis dynamics can be mediated with as few as two components. The non-adaptive dynamics mediating taxis responses provide an explanation for experimental observations made in mutant strains of *E. coli* and in wild-type *Rhodobacter sphaeroides* that could not be explained with standard models. We speculate that such dynamics exist in other bacteria as well and play a role linking the metabolic state of the cell and the taxis response. The simplicity of mechanisms mediating such dynamics makes them a candidate precursor of more complex taxis responses involving adaptation. This study suggests a strong link between stimulus conditions during evolution and evolved pathway dynamics. When evolution was simulated under conditions of scarce and fluctuating stimulus conditions, the evolved pathway contained features of both adaptive and non-adaptive dynamics, suggesting that these two types of dynamics can have different advantages under distinct environmental circumstances.

## Author Summary

Here, we study how signalling networks mediating chemotaxis could have evolved. We simulated the evolution of virtual bacteria, which can explore their environment by alternating between swimming and tumbling. The tumbling frequency is dictated by the output of a signalling network that senses extracellular nutrient levels, while the bacteria's reproductive success is determined by their ability to find nutrients. Under conditions of abundant food, we find that bacteria quickly evolve signalling networks that enable effective chemotaxis, where increasing nutrient levels increase tumbling frequency. Our findings provide explanation for network dynamics underlying similar behaviour as observed in certain mutant strains of *Escherichia coli* and in other bacterial species. Conversely, wild-type *E. coli* respond to increasing nutrient levels by decreasing their tumbling frequency and adapting to constant attractant levels. We observe such adaptive network dynamics when we repeat evolutionary simulations under conditions of scarce food. These findings suggest that (i) adaptation is not necessary for effective chemotaxis, (ii) an ancestral minimal chemotaxis system could have used a simple coupling between the signalling network and the metabolic state, and (iii) environmental conditions are one of the determining factors for the evolution of adaptive responses.

## Introduction

Bacterial responses to external stimuli and the pathways with which they are mediated are model systems for studying the molecular basis of behaviour. Much of the research in this field has focused on chemotaxis, the ability of bacteria to swim up a gradient of a chemical attractant, as performed in the model organism *Escherichia coli*. More than 30 years after the first studies [1],[2], we now have extensive knowledge of the underlying biochemical pathway [3],[4]. Briefly, *E. coli* swims in a forward direction (undergoing some degree of rotational diffusion) when the reversible motor proteins on its outer membrane rotate counter-clockwise (CCW) and the attached flagella intertwine to form an effective propeller. When the motors reverse and rotate clockwise (CW), the flagella disassociate and cause the bacterium to tumble, resulting in a new swimming direction. The switching frequency of the motor is coupled to receptor activity by a set of proteins constituting a signalling pathway. With increasing attractant levels, the excitatory branch of the pathway causes suppression of CW rotation and tumbling, while the adaptation branch causes the cell to resume its original tumbling levels at constant attractant concentrations independently of this concentration level. The former branch involves the receptor-coupled kinase CheA and the associated response regulator CheY, which when phosphorylated binds the motor and increases the probability of CW rotation. The adaptation is achieved via control of receptor methylation, and hence receptor sensitivity, through the proteins CheR and CheB. The combination of these two branches results in the tumbling frequency approximately following a negative time-derivative of the attractant concentration [5]. Adaptation is the hallmark of this response, allowing bacteria to perform temporal comparisons of attractant with high sensitivity over a wide dynamic range [5]–[9].

While this ‘*E. coli* paradigm’ of chemotaxis is well established, our knowledge of taxis responses towards other stimuli and in other species [10]–[12] indicates that the derivative response with adaptation observed in *E. coli* is neither universal nor necessary for effective taxis. For example, the response to oxygen in *E. coli* is believed to be mediated by the receptor Aer that lacks methylation sites, indicating lack of adaptation [13]. In *Rhodobacter sphaeroides*, adaptation to persistent stimuli occurs much slower or not at all [14]. In the same species, growth under aerobic conditions results in an ‘inverted’ chemotaxis response where increasing attractant concentration causes an increase in tumbling frequency [15]; this inverted response is also observed in certain Halobacteria [16] and in certain mutant strains of *E. coli* that have been ‘gutted’ of some or most of the chemotaxis proteins [17],[18]. Interestingly, these natural and mutant strains all still show the ability to chemotax.

These diverse chemotaxic dynamics could result from multiple pathways that allow bacteria to integrate information about the internal and external state to produce coherent taxis behaviour [13]. For instance, in *R. sphaeroides*, genetic studies indicate that the large number of taxis proteins in this species are arranged into several distinct pathways [19]. The chemotaxic response towards certain molecules in this species requires transport into the cell [20], demonstrating the link between metabolism and chemotaxis. Despite much effort, we still lack a comprehensive understanding of the different molecular mechanisms involved in bacterial taxis responses, their underlying dynamics, and how these different dynamics are integrated.

Here, we use a computational approach to address some of these questions by simulating the evolution of a taxis response using computer modelling of bacterial movement and pathway dynamics. These simulations use a population of virtual bacteria existing in a virtual world complete with a stimulus source that is assumed to signal the presence of favourable conditions. Bacteria start with a set of non-interacting proteins, as well as a receptor and a reversible motor. Interactions between the proteins evolve through random mutations, with bacteria selected for reproduction based on their ability to localise at sites of favourable conditions. These evolutionary simulations consistently result in bacteria with a strong ability to move towards the stimulus. Interestingly, under conditions of abundant stimuli these bacteria evolve so that the tumbling probability is directly coupled to stimulus levels without any adaptation. We find that such non-adaptive dynamics can be mediated by as few as two signalling components, allowing for the possibility of metabolites acting as effectors. Simulation conditions mimicking environments with scarce or fluctuating stimulus sources result in evolution of pathways with complicated dynamics that have features of both non-adaptive and adaptive dynamics. Combined with experimental observations, these results demonstrate that adaptive dynamics are not necessary for effective chemotaxis. This work also suggests that non-adaptive dynamics underlie the chemotaxis observed in gutted *E. coli* strains and may have a role in the complex taxis behaviour of *R. sphaeroides*. We speculate that mechanisms leading to such dynamics exist in current-day bacteria and provide a way to fine-tune taxis responses in different conditions, or link it to the energy state of the cell.

## Results

In order to study the evolution of bacterial taxis, we use virtual bacteria that move in a computer-based two-dimensional environment containing a fixed stimulus source and periodic boundary conditions. As such, this computer environment mimics a natural environment with abundant stimuli. The movement of these bacteria is coupled to the dynamics of a signalling pathway consisting of several proteins that catalyse each other's activation and deactivation, corresponding to kinases and phosphatases in real cells (see Methods). These proteins include a receptor whose activity level is directly coupled to the local stimulus level and an effector that, when activated, can bind to a reversible motor, reversing its direction and causing the bacteria to tumble. Evolutionary simulations start with a population of bacteria, each of which contains a certain number of proteins that are initially non-interacting. These bacteria are allowed to explore the environment for a ‘generation-time’ consisting of a certain number of time steps. At each time step, the bacteria either can continue to swim forward or can tumble to orient to a new random direction. Additionally, the concentrations of activated proteins in the pathways of each bacterium are updated, and the probability of tumbling during the next time step is computed based on the concentration of activated effector. After this generation-time, bacteria are selected for replication based on the integrated amount of stimulus they have encountered. During replication, there is a probability for mutations to occur, which alters the structure and parameters of the biochemical pathway. To summarise, these evolutionary simulations couple mutational events occurring at molecular level (i.e. pathway level) with selection at behavioural level (i.e. taxic response). Note that this is a generic model where the stimulus represents anything capable of activating a receptor (e.g., chemical attractant, light, pH) and there are no *a priori* assumptions regarding pathway structure or dynamics.

**Figure 1:**
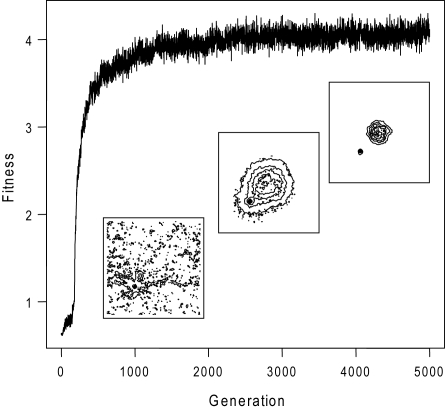
Evolution of the taxis response in silico. The average fitness in an evolving population of virtual bacteria. The inset shows the time-averaged distribution of positions of the population at generation 0, 200 (corresponding to a fitness of approximately 2.0), and 5,000 (final generation) as a contour plot. Areas enclosed by darker lines indicate more time spent there. Note that in these simulations the entire population starts at grid location (30,30) while stimulus source is fixed at (50,50).

**Figure 2:**
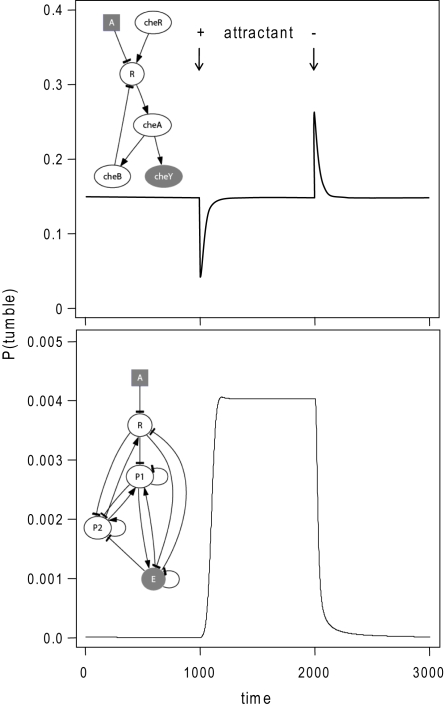
Adaptive versus non-adaptive pathway dynamics. Time course of phosphorylated CheY concentration (top), as simulated by the model presented in [10] and the time course of active effector concentration for the most frequent pathway in the evolutionary simulation described in Figure 1 (bottom). The inset shows the cartoon representation of this pathway. In both simulations, the system is allowed to pre-equilibrate for 1,000 timesteps. A stimulus of one is then added at time 1,000 and removed at time 2000. Kinetic parameters for the shown pathway are given in Dataset S1.

Figure 1 shows the population average of fitness (encountered stimuli) during one evolutionary simulation for a signalling pathway consisting of four proteins. As shown, the fitness value rapidly improves over a few generations and reaches a plateau. Clearly, the pathway structure and dynamics in virtual bacteria are evolving in such a way to mediate taxis. This behaviour can be seen from the average time spent by the population at different parts of the environment (see insets of Figure 1). While un-evolved bacterial populations are distributed irrespective of stimulus source, final populations are able to quickly co-localise with it. This behaviour is mediated by a specific biochemical pathway dynamics; at steady state, in absence of any signal, the concentration of activated effector is at a low level and the bacterium mostly swims without tumbling (see Figure 2 for typical pathway structure and dynamics, kinetic parameters are shown in Dataset S1). When the bacterium encounters higher stimulus levels, the effector is rapidly activated and stays activated as long as the signal is present, resulting in increased bacterial tumbling. We find that the qualitative nature of this type of dynamics is independent of basal stimuli level (data not shown). This non-adaptive dynamics allow the bacteria to spend more time in regions of high stimulus and swim straight when the stimulus level decreases. In evolutionary simulations repeated five times for pathways of 2 to 5 proteins, this mechanism always evolved as the dominant one.

The structures of pathways resulting from these simulations were diverse (see Dataset S1) indicating that there are several possible biochemical signalling cascades that can mediate non-adaptive dynamics. In case of the sample pathway shown in Figure 2, we find that the receptor acts as a global inhibitor shutting down effector activity in absence of stimuli. Incoming signals suppress receptor activity, allowing a build up of effector, which is involved in a feedback loop with one of the intermediary proteins, protein one (see cartoon representation in Figure 2). The other protein acts as a kinase (i.e. activator) on both the receptor and protein one, thereby ensuring rapid response termination when the stimulus is removed. This complex pathway structure and the resulting dynamics allow efficient chemotaxis behaviour as described above. However, similar dynamics can be achieved with much simpler circuits containing only two proteins (see Discussion).

Using a simple analytical model, we can capture the movement of bacteria as mediated by non-adaptive dynamics (see Methods). This model shows that in simple environments the presented dynamics should lead to bacteria accumulating approximately proportionally with the local level of the stimulus. Note that as long as the stimulus levels are above a certain threshold, this mechanism is only sensitive to the ratio of the relative levels and not their absolutely magnitudes. This suggests that an efficient taxis response can be achieved over a wide dynamic range of stimuli with pathway-dynamics that does not display adaptation to stimulus and results in increases in tumbling probability with increasing stimulus. Both these dynamical features are in striking contrast to the chemotaxis behaviour of *E. coli*, where the pathway ensures decreasing tumbling probability with increasing stimulus followed by rapid adaptation [5] (see Figure 2).

There are a number of different possible explanations for why the taxis pathways evolved in these simulations are characterised by an ‘inverted’ response (i.e. response to increasing stimuli is opposite of that seen in *E. coli*) and non-adaptive dynamics. Firstly, the evolutionary processes as modelled here might make non-adaptive pathways more evolutionarily accessible. Secondly, it might be that the modelled environmental situations are particularly well-suited for taxis mediated via such dynamics. In particular, the assumptions of stimulus consistency and abundance in the environment might reduce the need for adaptation. Thirdly, there could be other factors such as intra-cellular communication, multi-state receptors (i.e. receptors with methylation sites), and various physical processes [21] that are not included in the model and that could be important for the evolution of taxis responses mediated by other dynamics.

To see if the non-adaptive dynamics were the result of the difficulty of evolving adaptive dynamics, we performed additional simulations. These started with an initial bacterial population containing biochemical pathways with dynamics similar to that found in *E. coli* [22]. In five separate simulations, the bacteria always evolved more fit pathways with non-adaptive dynamics and inverted response. In other words, under the conditions of these simulations (i.e. under high stimulus abundance), there always existed a pathway with non-adaptive dynamics that could mediate a more efficient taxis response than the original adaptive pathway. This indicates that the results we obtain are not due to lack of an evolutionary route to the conventional dynamics observed in *E. coli*. It does not indicate, however, that taxis responses mediated by non-adaptive dynamics are superior as it was not possible to reproduce all environmental conditions and the other possibly-important features as mentioned above.

**Figure 3:**
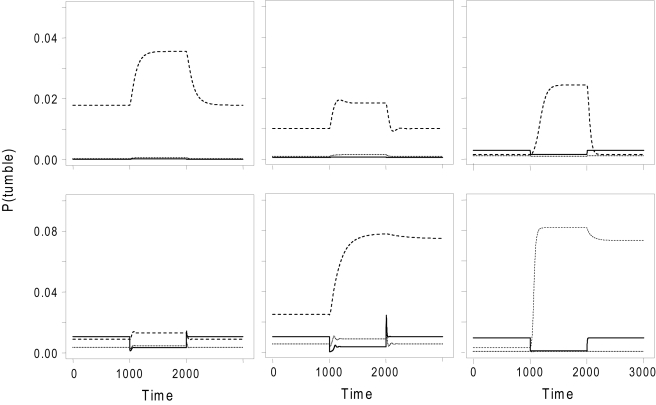
Diverse taxis mechanisms. Time course of active effector concentration for the most frequent and unique pathways obtained from selected evolutionary simulations that are run under different environmental conditions (see main text). Each panel displays dynamics for a specific pathway structure shown in the inset. The system is allowed to equilibrate for 1,000 timesteps at the background basal stimulus level. A stimulus of one is added at time 1,000 and removed at time 2,000. Pathway response to such stimuli given on top of a selected basal level is shown in different line types (basal level is 0, 1, and 2, respectively, for solid, dotted, and dashed). Pathways shown in the upper panels are from simulations with periodic boundary conditions and fluctuating stimulus source, while those shown in the lower panel are from simulations with non-periodic boundary conditions and fixed stimulus source. Kinetic parameters for these pathways are given in Dataset S1.

To explore the effect of environmental conditions on the evolution of chemotaxis, we ran two sets of simulations under (i) periodic-boundary conditions and fluctuating stimulus source and (ii) non-periodic boundary conditions and fixed stimulus source. The first set of conditions allow us to test the hypothesis that adaptive dynamics provide a means for bacteria to preserve robustness of the response to fluctuations in the external environment or internal parameters [23]. The latter conditions mimic an environment with scarce stimulus, where exploration is expected to be more important than exploitation. In five simulations run under each condition, we did not find pathways with dynamics that are adaptive over a wide range of stimuli as seen in *E. coli*. However, several simulations resulted in pathways that had dynamical behaviour similar to that of *E. coli* under some conditions. As shown in Figure 3, these pathways give a “normal” response (i.e. decreased tumbling probability with stimuli) and have limited adaptation to continuous stimuli. Interestingly, most pathways evolved under non-periodic boundary conditions show dynamics that are dependent on basal stimuli levels. This affects mostly the adaptation dynamics and we observe one pathway achieving perfect adaptation under a narrow range of basal stimuli levels (see Figure 3). Most simulations run under sparse stimulus conditions resulted in approximately same fitness levels as shown in Figure 1. However, simulations run under these conditions (ii) took much longer (usually more than 2000 generations) to reach these fitness levels. Taken together, these results indicate that realistic and complex environmental conditions lead to evolution of complex pathway dynamics that contain features of both adaptive and non-adaptive dynamics. Untangling the role of each type of dynamics in the efficiency of chemotaxis requires further detailed analyses.

## Discussion

The molecular systems mediating the taxis responses observed in bacteria are more complicated than the dominant picture of *E. coli* chemotaxis suggests. Bacteria can sense and respond to a variety of environmental clues, possibly integrating the signal from different biochemical pathways. Recent experimental observations from an increasing number of bacterial species and past studies from mutant strains of *E. coli* hint at the diversity of molecular mechanisms involved in generation of these responses. Here we provide evidence for the effectiveness of one possible dynamical scheme. The main features of this dynamics is an inverted response, leading to increasing tumbling frequency with increasing stimulus level, and an absence of adaptation to continuous stimuli. We show that such non-adaptive dynamics readily evolve under different environmental conditions and model assumptions and allow bacteria to accumulate at favourable conditions efficiently.

**Figure 4:**
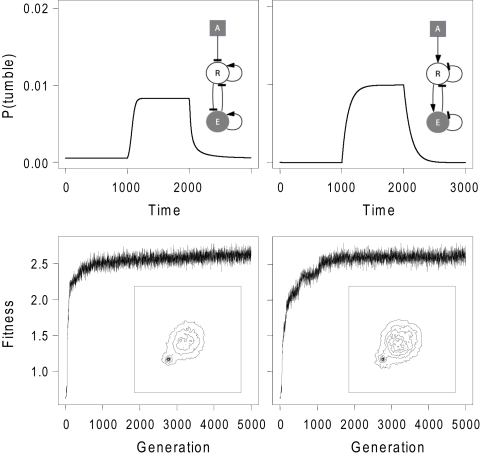
Minimal pathways with non-adaptive dynamics. Cartoon representation and time course of active effector concentration for the most frequent and unique pathways with 2 proteins obtained from 5 different evolutionary simulations. The pathway on the left and right were found in 1 and 4 simulations, respectively. Bottom panels show fitness curves for the corresponding simulations and the time-averaged distribution of positions of the final population. Kinetic parameters for these pathways are given in Dataset S1.

These findings provide a possible explanation for the non-adaptive dynamics and inverted responses observed in wild type *R. sphaeroides* [14],[15] and the inverted responses observed in Halobacteria [16] and gutted strains of *E. coli* [18]. In each case, efficient taxis responses were observed, although the exact nature of the underlying molecular mechanisms could not be determined. It is likely that these mechanisms form systems similar to the pathways presented here. An analysis of results from simulations with two proteins reveals the minimum signalling systems to achieve taxis responses mediated by non-adaptive dynamics (see Figure 4). They involve coupling of the signal to an effector via a receptor, with self-regulation of both proteins (through allosteric interactions or processes such as auto-phosphorylation). The striking simplicity of these minimal systems lead to the speculation that non-adaptive dynamics could even be achieved without any signalling proteins; a small molecule, that is a by-product of metabolism or is taken into the cell via a transporter, could directly regulate tumbling probability of the cell. We hypothesise that exactly such a scenario is responsible for chemotaxis observed in gutted *E. coli* [18]. Attractant-related metabolism causes increases in fumarate levels inside the cell, which binds the motor and increases tumbling probability. It has been demonstrated experimentally that fumarate can be involved in chemotaxis [24] and can control motor switching [25], although the exact dynamics of how it could lead to chemotaxis was unknown.

If non-adaptive dynamics are available and provide efficient taxis responses, why do we observe adaptive dynamics in *E. coli* and other bacterial species? Adaptive mechanisms might be more efficient in exploring the environment and achieving a robust response under fluctuating stimuli. The evolution of complex dynamics in simulations run under conditions mimicking scarce and fluctuating stimulus sources supports such arguments. The dynamics of these pathways contained both adaptive and non-adaptive features, further indicating the possible complexity of chemotaxis behaviour. As indicated by experiments with gutted *E. coli* and other species, non-adaptive pathways probably function in conjunction with the canonical mechanism, and are involved in the fine-tuning of taxis responses under certain environmental conditions or in providing a link between energy related taxis responses and chemotaxis. Alternatively, given the simplicity of the required molecular machinery, the non-adaptive dynamics could be the precursor of the more complicated adaptive mechanisms. Both hypotheses could be tested with specific experimental setups and a sequence analysis of the proteins involved in taxis responses respectively.

## Methods

To study the pathways underlying taxis responses we used a previously described pathway model [22]. This model assumes that a pathway consist of a set of *N_p_* proteins, all of which can exist in a deactivated or activated state (activation can correspond to phosphorylation, methylation, or any other type of chemical or structural modification). Each protein is capable, in the activated state, of causing the activation or deactivation of any of the other proteins. The first protein in the pathway is arbitrarily chosen to act as a receptor that can be activated by the external stimuli, while protein *N_p_* is arbitrarily chosen to be an effector. When activated, it can bind to the motor protein causing a reversal of the motor and the bacterium to tumble. The biochemical dynamics for [P_i_*], the fraction of protein *i* that is activated, obeys
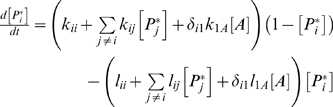
(1)where *k_ij_* (*l_ij_*) is the rate at which activated protein *j* activates (deactivates) protein *i*, *k_ii_* (*l_ii_*) is protein *i*'s rate of self-activation (deactivation), *k_1A_* (*l_1A_*) represents the rate at which the stimulus activates (deactivates) the receptor protein 1, [*A*] is the local level of stimulus, and δ is a Kronecker delta. The total concentration of each protein is assumed to be one. For simplicity we assume that any given protein can either activate or deactivate another, but not both. That is, *k_ij_ l_ij_* = 0 for all *i* and *j*. We formulate this by considering a value of γ*_ij_*, where positive (negative) values of γ*_ij_*, correspond to positive values of *k_ij_* (*l_ij_*). This can be expressed as
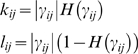
(2)where *H(x)* is the Heaviside step function, equal to one if *x* is positive, and zero otherwise. The probability *p*_Tumble_ of a protein tumbling at any given timestep is given by
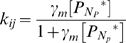
(3)where γ*_m_* is the affinity of protein *N_p_* for the motor.

The evolutionary simulations are carried with a population of *N*_bacteria_ = 1000 bacteria existing on a two-dimensional 100×100 square space with periodic boundary conditions. The level of stimulus is defined as a Gaussian distribution located at the centre (50, 50) of the space with maximum value 10.0 and width 7.1. At each generation, every bacterium starts at a location (30, 30) pointed in a random direction, with all activated protein concentrations set to zero. At each timestep the bio-kinetic equations (Equation 1) are integrated based on the local level of stimulus. The bacterium then can either tumble (choose a new random direction) with probability *p*_Tumble_ or, alternatively swim in a straight line in the current direction a distance given by the swimming speed (0.2). Each bacterium absorbs an amount of stimulus proportional to the local stimulus level, without consuming it. At the end of *N*_step_ = 5000 timesteps, the next population of bacteria are selected using tournament selection: a set of five bacteria is chosen at random, and of the five, the bacterium that has accumulated the most stimuli is assigned to the next generation. This procedure is repeated (with replacement) *N*_bacteria_ times. During each assignment process, there is a probability *p*_mutation_ = 0.1 of a mutation occurring. This mutation involves adding or subtracting a Gaussian-distributed value (mean 0, standard variation 0.1) to one of the γ*_ij_* chosen at random.

In summary, the resulting pathway model captures the basic biochemistry of signalling pathways and allows coupling of external signals to the tumbling probability. The basic assumptions of the model are that proteins can only occur in two states and that each protein can interact with any other. The latter assumption allows generality in the model without imposing limitations. Every pathway structure that could be constructed in the presented model could be constructed with real biochemistry (potentially requiring more proteins). Still, we have tested the effects of imposing possible limitations on the pathway structure on the evolution of taxis responses. These included imposing the requirement that the receptor can only be a kinase (i.e. it could only activate other proteins), that each protein can have only a single interaction, or that self-regulation is not allowed in the model. We find such limitations not to significantly affect the outcome of the evolutionary simulations (data not shown).

To test the effect of having fluctuating stimulus source in the environment on the evolution of taxis responses we run additional simulations where a number of the parameters were chosen independently for each generation. These included; (i) a background uniform stimulus distribution, chosen from a uniform distribution [0–5.0], (ii) the peak height of the Gaussian stimulus distribution, chosen from a uniform distribution [1.0–10.0], (iii) the variance (width squared) of the Gaussian stimulus distribution, chosen from a uniform distribution [50.0–500.0], and (iv) the initial location for the bacteria chosen at random from all points in the 100×100 space. In addition, for these runs, 20 percent of all mutations resulted in an interaction being deleted (set equal to zero) rather than modification through addition of a randomly chosen increment. The result of these simulations were analysed by subjecting the pathway to a specified time-course of stimulus levels, and monitoring the resulting response as shown in Figure 2.

To perform a simple estimate of the efficiency of taxis response mediated by the non-adaptive pathway dynamics (see main text), we consider the equilibrium situation where there is a steady-state concentration of bacteria 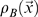 and stimulus 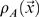 at any location 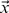. At each time step a bacterium can either tumble or swim, so we can express the probability that a bacterium that starts at location 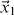 will swim to location 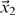 as equal to 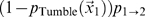, where *p*_1→2_ is the probability that a swimming bacterium at location 1 will swim to location 2. If we now invoke detailed balance, the overall flux of bacteria swimming from 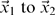 will exactly equal those swimming from 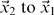, or 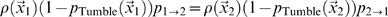. In general, *p*_1→2_ will be a complicated function that includes information about the previous location (and thus the current swimming direction) of the bacterium at 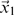, but if we ignore these correlations and assume that the bacteria are swimming in an isotropic manner in a simple space, *p*_1→2_ = *p*_2→1_. We can further assume that for the described non-adaptive pathway dynamics, the concentration of the effector is proportional to the local stimulus level 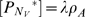. Under these conditions, assuming the tumbling probability follows Equation 3, it is straightforward to show that the relative concentration of bacteria at different locations is given by 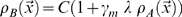 where *C* is a normalisation constant. In other words, for sufficient gain (γ*_m_* λ) and stimulus level, the steady-state bacteria concentration is proportional to the stimulus level resulting in an efficient taxis response.

## Supporting Information

Dataset S1 Most frequent and unique pathways resulting from all the evolutionary runs explained in the main text.
Pathway structure is shown in a matrix form, where each row lists the coefficient of interaction between a given protein and all the others. Letters, A, R, P, and E stand for attractant, receptor, protein, and effector respectively. Note that attractant can only interact with the receptor. The affinity of the effector for the motor (γm) is shown next to the interaction matrix. The coefficients for each pathway are used to simulate its dynamics as described in Methods. Matrices, for which the dynamic behavior is shown in the main text are: Figure 4: All shown matrices for 2-protein pathways, fixed attractant conditions. Figure 2: The matrix from run 03 for 4-protein pathways, fixed attractant conditions. Figure 3 top row: Matrices from runs 02, 03, and 04 for 4-protein pathways, fluctuating attractant conditions. Figure 3 bottom row: Matrices from runs 02, 03, and 04 for 4-protein pathways, non-periodic boundary conditions.
(0.46 MB DOC)
