# Supplementary material for: Evolution of Taxis Responses in Virtual Bacteria: Non-Adaptive Dynamics
Source: PLoS Comput Biol. 2008 May 23;4(5):e1000084. doi: 10.1371/journal.pcbi.1000084 (PMC2386285; doi:10.1371/journal.pcbi.1000084)
Supplement: Dataset S1 — Most frequent and unique pathways resulting from all the evolutionary runs explained in the main text. Pathway structure is shown in a matrix form, where each row lists the coefficient of interaction between a given protein and all the others. Letters, A, R, P, and E stand for attractant, receptor, protein, and effector respectively. Note that attractant can only interact with the receptor. The affinity of the effector for the motor (γm) is shown next to the interaction matrix. The coefficients for each pathway are used to simulate its dynamics as described in Methods. Matrices, for which the dynamic behavior is shown in the main text are: Figure 4: All shown matrices for 2-protein pathways, fixed attractant conditions. Figure 2: The matrix from run 03 for 4-protein pathways, fixed attractant conditions. Figure 3 top row: Matrices from runs 02, 03, and 04 for 4-protein pathways, fluctuating attractant conditions. Figure 3 bottom row: Matrices from runs 02, 03, and 04 for 4-protein pathways, non-periodic boundary conditions. (0.46 MB DOC) [file pcbi.1000084.s001.doc]

Most frequent and unique pathways resulting from all the evolutionary runs explained in the main text. Pathway structure is shown in a matrix form, where each row lists the coefficient of interaction between a given protein and all the others. Letters, A, R, P, and E stand for attractant, receptor, protein, and effector respectively. Note that attractant can only interact with the receptor. The affinity of the effector for the motor (γ*_m_*) is shown next to the interaction matrix.

The coefficients for each pathway are used to simulate its dynamics as described in *Methods*. Matrices, for which the dynamic behavior is shown in the main text are:

Figure 4: All shown matrices for 2-protein pathways, fixed attractant conditions.

Figure 2: The matrix from run 03 for 4-protein pathways, fixed attractant conditions.

Figure 3 top row: Matrices from runs 02, 03, and 04 for 4-protein pathways, fluctuating attractant conditions.

Figure 3 bottom row: Matrices from runs 02, 03, and 04 for 4-protein pathways, non-periodic boundary conditions.

2-protein pathways, fixed attractant conditions:

3-protein pathways, fixed attractant conditions:

4-protein pathways, fixed attractant conditions:

5-protein pathways, fixed attractant conditions:

4-protein pathways, fluctuating attractant conditions:

4-protein pathways, fluctuating attractant conditions, and starting with matrix:

4-protein pathways, non-periodic boundary conditions:
